# Zoonotic parasites associated with predation by dogs and cats

**DOI:** 10.1186/s13071-023-05670-y

**Published:** 2023-02-06

**Authors:** Jairo Alfonso Mendoza Roldan, Domenico Otranto

**Affiliations:** 1 Department of Veterinary Medicine, University of Bari, Valenzano, Italy; 2 Faculty of Veterinary Sciences, Bu-Ali Sina University, Hamedan, Iran

**Keywords:** Predation, Parasites, Dogs, Cats, Zoonoses, Intermediate hosts, Paratenic hosts, Reptiles, Birds, Rodents

## Abstract

One of the most common behaviors of cats that have an indoor/outdoor lifestyle is to bring hunted “gifts” to their owners, represented by small mammals, reptiles and birds. Access to the outdoors by dogs and cats may represent a problematic issue, since they may be at risk of diseases, traffic accidents and ingestion of toxins. Yet, the impact of this population of roaming dogs and cats predating wildlife is another concerning issue that receives less attention. Despite these risks, most owners still prefer to give outdoor access to their pets to allow them to express their “natural instincts,” such as hunting. Therefore, with a growing population of > 470 million dogs and 373 million cats worldwide, predation not only represents a threat to wildlife, but also a door of transmission for parasitic diseases, some of them of zoonotic concern. In this review, the role played by dogs, and especially cats, in the perpetuation of the biological life cycle of zoonotic parasites through the predation of rodents, reptiles and birds is discussed. Feral and domestics dogs and cats have contributed to the population collapse or extinction of > 63 species of reptiles, mammals and birds. Although the ecological impact of predation on wild populations is well documented, the zoonotic risk of transmission of parasitic diseases has not received significant attention. The parasitic diseases associated to predation vary from protozoan agents, such as toxoplasmosis, to cestodes like sparganosis and even nematodes such as toxocariasis. Raising awareness about predation as a risk of zoonotic parasitic infections in dogs and cats will aid to create responsible ownership and proper actions for controlling feral and free-roaming cat and dog populations worldwide.

**Graphical Abstract:**

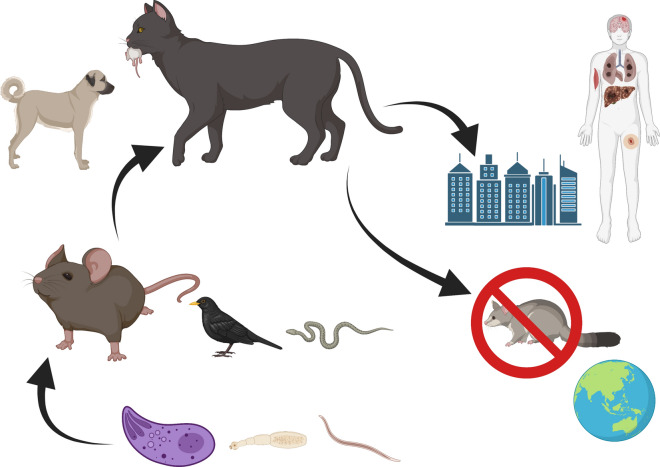

## Background

To a cat owner, waking up in the morning or arriving home after an exhausting day at work just to find that “Milo” is playing in bed or near the kitchen with a half-dead lizard is an unpleasant event that may not be so uncommon as many would like it to be. Indeed, cats are known to not just hunt small reptiles, rodents and birds, but also to bring to their owners some of the day's hunt as a “gift” [1, 2]. In fact, dogs, but especially cats, represent top predators that can adapt to virtually any type of environment, mostly near urban settlements, as well as feral populations of dogs and cats that continue to be on the rise in all continents [3, 4]. Domestic dogs and cats have been part of our society for > 10,000 years [5–7]. An emotional attachment to these species is evident in most of the western world and has created a more permissive culture toward behaviors that may have consequences in conservation and animal welfare, such as allowing animals to have unrestricted access to the outdoors [8, 9]. These behaviors are more evident in cats who are allowed to wander and have a partial to total outdoor life [10]. This lifestyle is debatable, and even today there is no consensus on whether cats should be allowed to go outside or not [11–13]. However, the devastating consequences of feral populations and outdoor dogs and cats produce in the decline of the native fauna is not debatable [14, 15]. Indeed, recent studies have shown the direct and high impact of predation by dogs and cats on populations of small mammals, birds, reptiles and amphibians [16–18]. Thus, it has been consistently advocated to avoid outdoor lifestyles and to reduce the feral dog and cat populations to decrease their impact on the native fauna as well as to avoid the further extinction of wild species [19–21]. Conversely, other problematics raised by predation have been scarcely discussed [22]. Indeed, predation is one of the most efficient strategies of parasite transmission, as it is a direct way for a parasite to complete its life cycle, depending on the trophic chain [23, 24]. Trophic-transmitted parasites have, in many cases, evolved strategies to enhance predation via prey manipulation [25]. For example, *Toxoplasma gondii* (Eucoccidiorida: Sarcocystidae) depends on the ability of feline predators to feed on small rodents and other prey species to complete its life cycle [26, 27]. Hence, one of the diseases that has been used as a paradigmatic example to avoid the predatory behavior of cats and dogs is toxoplasmosis, which is a highly prevalent parasite in cat and human populations [28, 29]. Therefore, in this review we discuss the parasitic diseases associated with predation, with a focus on those that are of zoonotic concern, to further evidence the risk of transmission of parasitic diseases associated to outdoor lifestyle of dogs and cats.

## *Greta* and *Valma* are top predators: origin of domestic feline and canine populations

The domestication process of dogs and cats was quite different and ultimately resulted in a diverse range of breeds, sizes and phenotypes of dogs but a more conserved and almost ancestral form of modern cats [30–33]. On one hand, the domestication of dogs occurred around 10,000 years ago, based on a need of primitive humans to hunt in packs, as wolves do [34, 35]. Thus, modern dogs originated from wolves, using empiric knowledge and soon after that of genetics to create breeds with different purposes (e.g. hunting, searching, company) [35]. On the other hand, domestication of modern cats is believed to had happen several thousands of years before the domestication of dogs [36, 37]. The relationship between cats and humans had a pest control purpose, aiding human populations in keeping the rodent and other vermin populations under control, eventually favoring the transformation of a hunting-based society to a farming, stable human population. Given this unique purpose of cats, besides being company to their owners, breeds of cats were created solely with an esthetic purpose, with more than 50 modern breeds of cats [38]. Despite this relatively large number of cat breeds, the modern cat morphology has not changed as dramatically as that of dogs. Furthermore, cats and hunting breeds of dogs still maintain their predatory instincts.

**Fig. 1:**
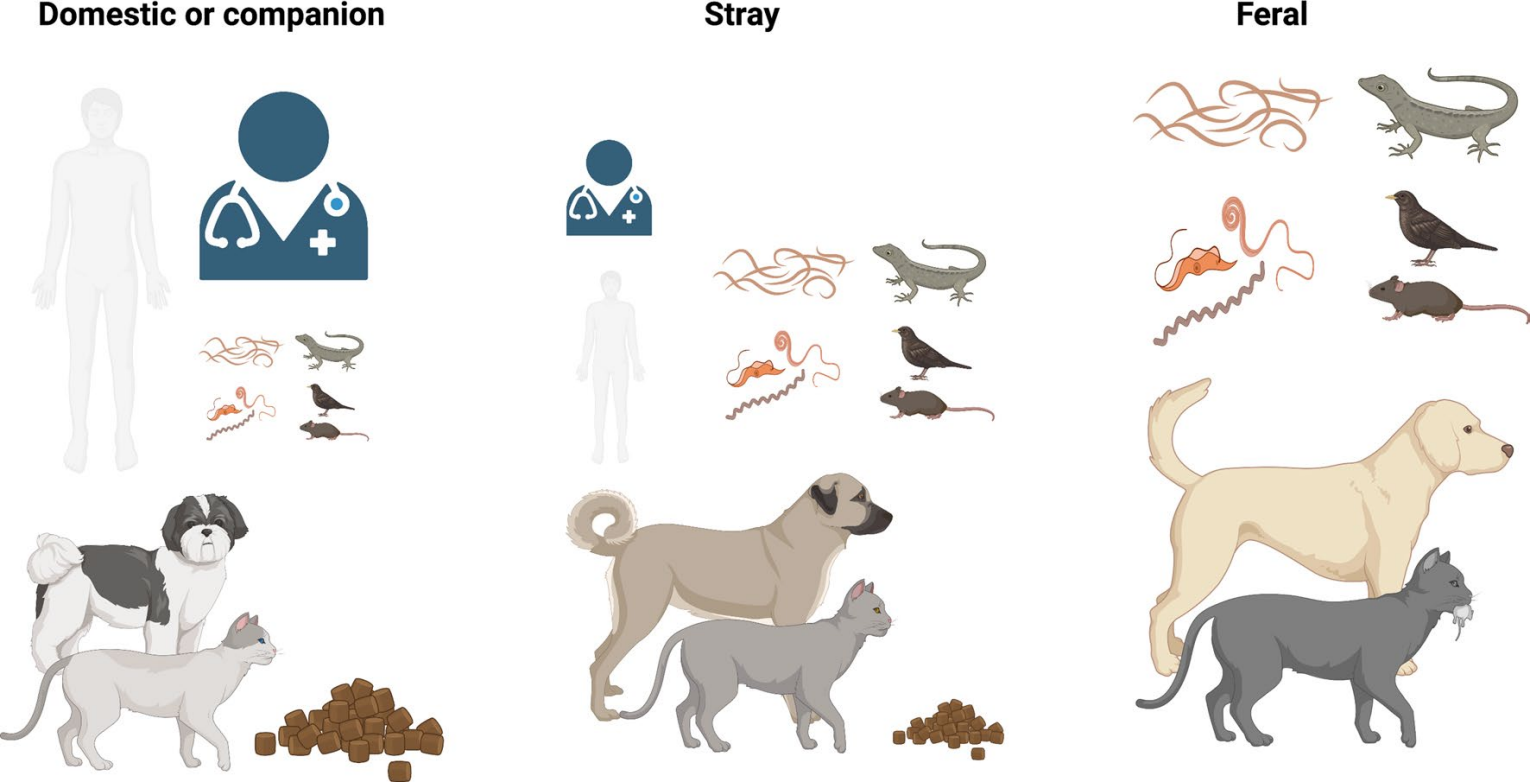
Categories of dog and cat populations according to contact with humans, healthcare access and exposure to parasites as a consequence of predation

Dogs and cats are a pivotal part of modern civilization, as more than half of the human global population is estimated to have a pet at home, dogs being the most popular, present in one of three homes worldwide [39, 40]. In addition, almost a quarter of pet owners have a cat [41]. For example, in a survey conducted in 2021–2022 in the USA, about 70% households (i.e. 90.5 million families) owned a pet, specifically 45.3 million cats and 69 million dogs, with a total pet industry expenditure of $123.6 billion [42, 43]. Apart from the growing domestic dog and cat population (estimated around 900 million and 600 million, respectively), a large portion of this number is represented by wild and feral animals, which make up to more than half of the total number [21, 44]. In addition, feral populations have a major negative impact on conservation and disease transmission [21]. Specifically, the dog and cat population can be classified in three main groups (Fig. 1): domesticated or companion animals, which are animals that are in tight contact with humans, receive proper husbandry and healthcare, have a lower burden of parasites and predate occasionally on small animals [45]. Stray animals are those that have occasional contact with humans. Thus, some of them receive less food and shelter, as well as healthcare, being more exposed to parasites and feeding mainly on small prey [46, 47]; feral animals are those that have no contact with humans and are independent. Thus, they do not receive food or healthcare, have a higher burden of parasites and feed mainly on small prey [48]. There is a large and ongoing debate on control policies for these “wilder” populations of dogs and cats [49, 50]. Regardless of this classification, cats have a more independent nature than dogs, which makes them more prone to hunt than stray or domesticated dogs. Moreover, dogs in general can be trained to reduce their hunting behavior, differently from cats, which have a very strong hunting instinct, which often is even encouraged by owners [51].

Given humanity’s emotional attachment to cats and dogs, the general appeal and tight relationship between dogs, cats and humans has hindered their population control, since reduction or elimination plans may be considered animal abuse [52–54]. Hence, policy makers and legislators have imposed softer control measures such as the Trap-Neuter-Return (TNR) program in the USA for cats or the outdoor cat colonies in many countries in Europe, completely prohibiting euthanasia as a control measure [55, 56]. Consequently, invasive cat and dog populations have had a devastating impact in places such as Galapagos Islands, Mauritius, Madagascar and Australia, to name a few [57–60].

## Predation and its impact beyond conservation

**Fig. 2:**
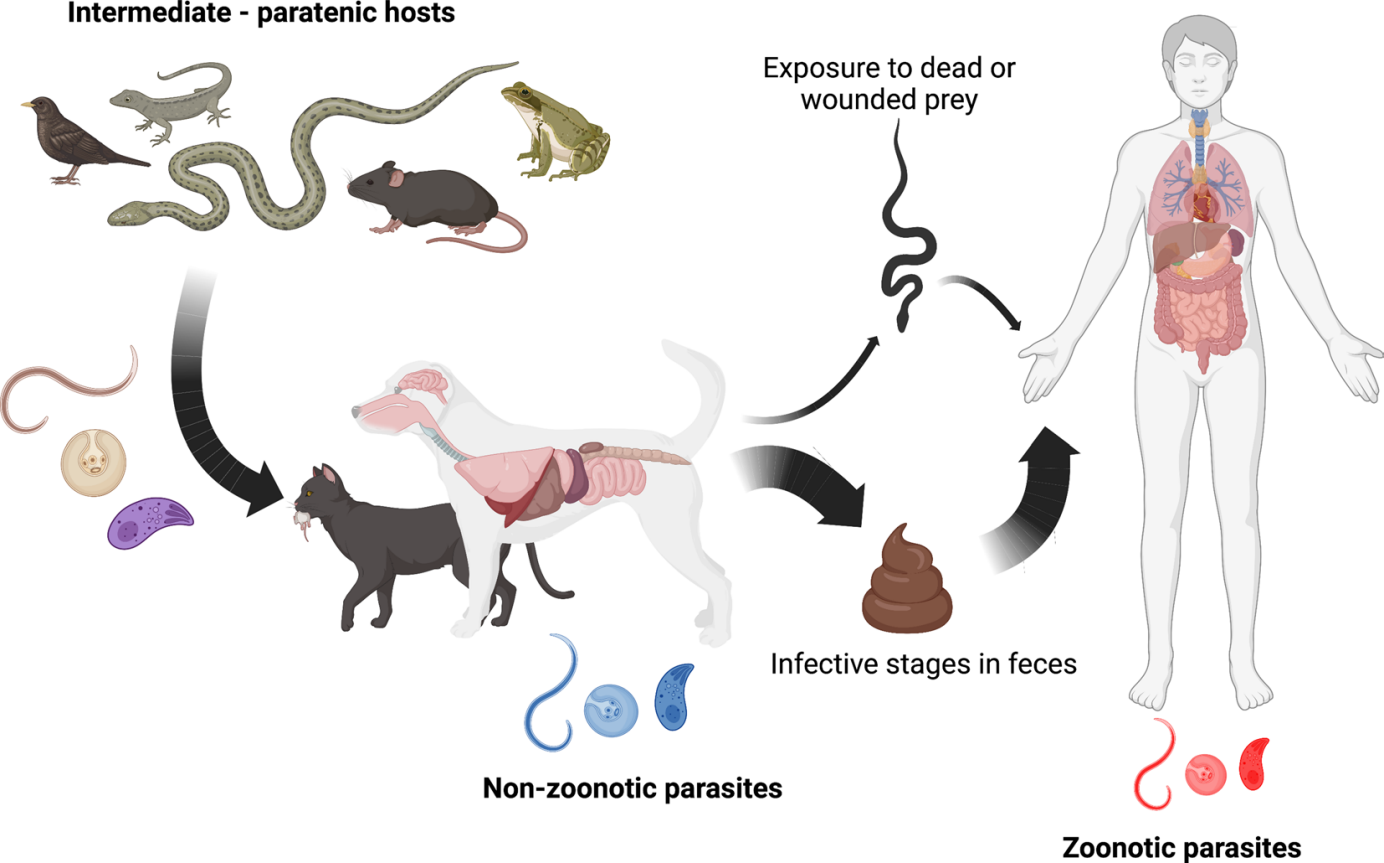
Transmission routes of zoonotic parasites of dogs and cats associated with predation

Dogs and cats, especially those with feral behavior, represent an important threat to biodiversity. However, predation also may be a gateway for the transmission of parasites, some of which may have zoonotic potential, threatening not only our companion animals but also human health [61, 62]. Despite this potential of zoonotic transmission, most efforts and studies have been focused on the impact of predation in conservation [63–66]. Indeed, many studies have correlated the presence of dogs and mainly cats with the decline or extinction of native populations. For example, cats were the partial cause of the extinction of the Stephens Island wren (*Traversia lyalli*) in New Zealand [67]. Likewise, cats have reduced the native fauna in many ecosystems and caused the extinction of small animals on islands [68–70]. The primary trophic source for dogs and cats is small mammals, birds, reptiles and invertebrates [71, 72]. Although feral populations contribute mainly to the predatory pressure on small prey, domestic dogs and cats with an outdoor lifestyle also have an important impact on endemic populations of wildlife, being also a source of zoonotic infection for human beings. For example, studies in suburban areas of the US demonstrated that > 44% of cats that had outdoor lifestyles preyed on small wild animals, with 23% of their prey being brought back to their owners [73]. Considering this, prey brought back to the household may represent another transmission route of parasites (Fig. 2), apart from fecal-oral transmission, more important than currently acknowledged [74].

**Fig. 3:**
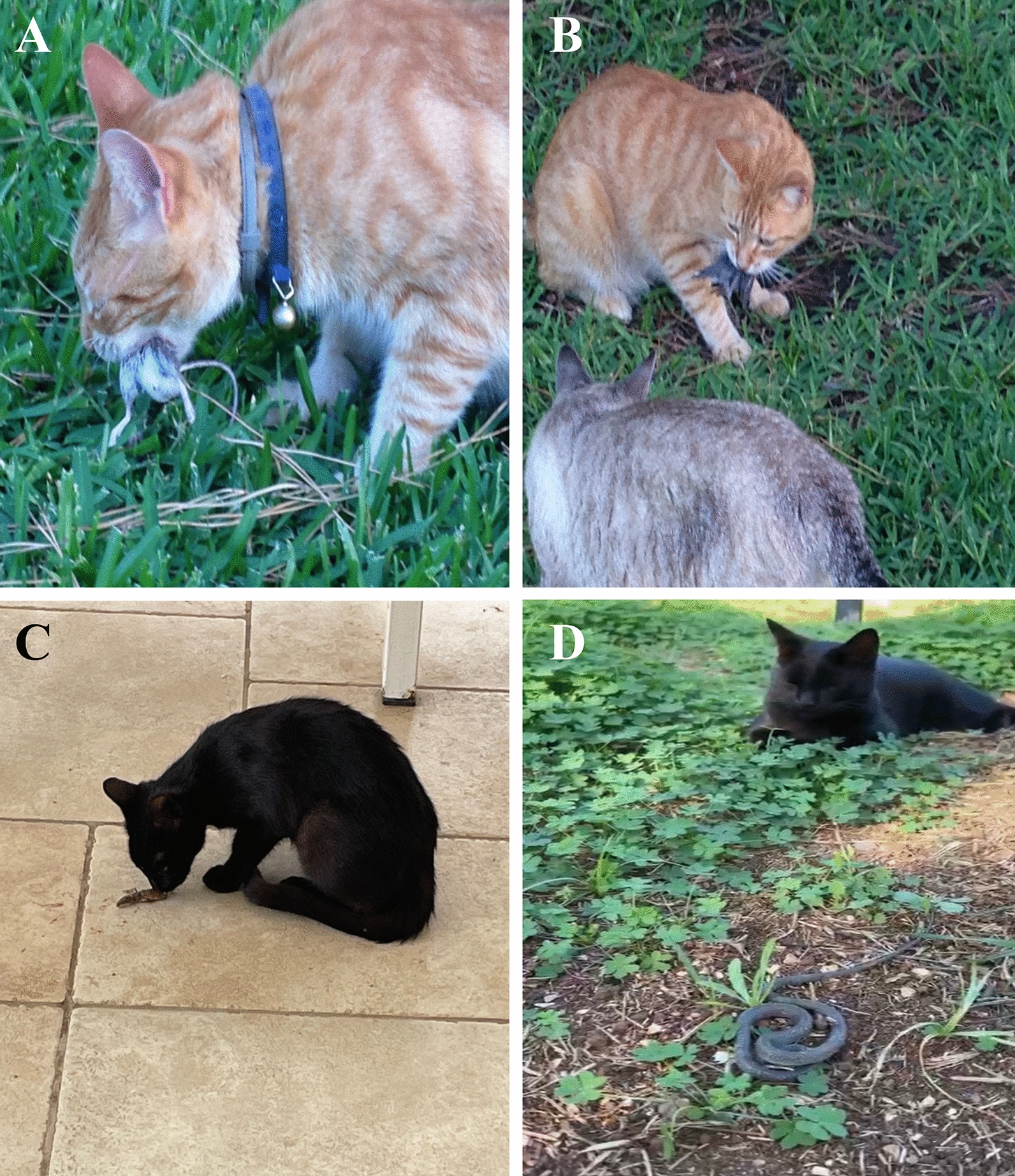
Cats predating small animals. **A** Cat hunting a mouse; **B** cats hunting a passerine bird; **C** cat eating a lizard; **D** cat near a mauled snake

The deleterious impact of predation has been particularly evident on islands such as in Australia and New Zealand, where no other predators were present; thus, the wildlife was more vulnerable to invasive predators, such as dogs and cats [75, 76]. Indeed, it is estimated that feral cats kill > 466 million reptiles per year in Australia, where a single cat may kill up to 225 reptiles per year [76]. However, the situation is not less worrisome elsewhere. Studies estimated that > 478 million reptiles are killed by cats in the US per year [16]. Conversely, the impact of dog predation has been less assessed and quantified. However, studies performed in Tasmania, showed that dogs also are a cause of native wildlife population decline and disturbance [77]. Dogs may also have a devastating effect on small and vulnerable populations, such as the complete annihilation of the largest flamingo colony in Sardinia, Italy [78] or the predation of 55.5% (500/900) of a kiwi bird population by a single dog in New Zealand [79]. Overall, dogs and cats are estimated to have caused the extinction of > 63 species of small animals (i.e. rodents, birds, reptiles and amphibians) (Fig. 3) [68]. However, of all the invasive species (e.g. red foxes, pigs, dogs, mongoose, wild boars), cats are the primary cause of population decline and extinction of endemic animals worldwide [80]. Therefore, though feral and colony cats are the main threat to vulnerable endemic species, especially on islands [57, 61, 64], their control and eradication are under debate because of the perception of “beneficial” predation of cats toward pest populations of mice and rats [81].

Overall, predation is a typical ancestral behavior of dogs and cats, which greatly affects many ecological processes, ultimately favoring parasites in the completion of their biological life cycle. Indeed, parasites not only regulate ecological processes, reducing host survival and fitness [82, 83], but also host abundance [84]. Nonetheless predation has long been overlooked in many fields of parasitology, such as parasite ecology, biology and epidemiology [85], resulting in lack of knowledge on the role prey may exert on the maintenance and control of parasitic diseases of dogs and cats. This is also due to the difficulties in studying intermediate and paratenic hosts in parasitology, which are mainly related to the long time required for running those studies under field conditions. Nevertheless, in this review we highlight and point out some parasites of zoonotic concern that are strictly related to predation as a strategy to complete their life cycle.

## Predation and zoonotic parasites

Many species of parasites (i.e. genera *Spirura*, *Physaloptera*, *Gnathostoma, Diplopylidium* or *Joyeuxiella*) use small animals, such as rodents, reptiles and birds, as intermediate or paratenic hosts and dogs and cats as definitive hosts. While most of these parasites are specific for their animal hosts, other are of zoonotic concern (Table 1) [62, 86, 87]. Recent extrinsic factors (e.g. environmental and climate modifications, urbanization, habitat fragmentation) have favored the trophic transmission of parasites [88, 89]. This has resulted in an increased risk of spill-over of parasites from wild to peri-urban and urban settings favoring the contact of predators and prey, along with the parasites they carry. For example, in Australia two zoonotic parasitic diseases associated with the presence of cats are toxoplasmosis and sparganosis, carried by *Spirometra* spp. (Cestoda: Diphyllobothriidae), which may overtly spill over to native fauna and human populations [90]. Indeed, toxoplasmosis, besides being of great public health concern, has contributed to the decline of native mammal and bird populations, such as the urban populations of the Eastern Barred Bandicoot (*Perameles gunnii*) [91]. The other disease highly prevalent in feral cats in Australia is sparganosis, which causes human infection and also affects native fauna [92].

**Table 1:** Zoonotic parasites of dogs and cats associated with trophic transmission

Parasite	Definitive host	Intermediate hosts	Distribution	Disease in humans
Protozoa				
* Toxoplasma gondii*	Cats	Rodents, birds	Cosmopolitan	Toxoplasmosis
* Sarcocystis nesbitti*	Cats, snakes	Monkeys, rodents	Malaysia	Acute muscular sarcocystosis
* Cryptosporidium* spp.	Dogs, cats	Rodents, lizards, amphibians	Cosmopolitan	Diarrhea
Cestoda				
* Mesocestoides lineatus Mesocestoides literatus*	Dogs, cats	Rodents, lizards, amphibians	Cosmopolitan	Mesocestoidiosis
* Echinococcus multilocularis*	Dogs, cats	Rodents	North America, Europe, Japan	Alveolar Echinococcosis
* Taenia serialis* *Taenia brauni*	Dogs, (rare in Cats)	Lagomorphs, rodents	Cosmopolitan (mainly in Africa)	Coenurosis
* Spirometra mansoni* * Spirometra erinaceieuropaei*	Dogs, cats	Fish, amphibians, snakes	Cosmopolitan	Sparganosis
Nematoda				
* Toxocara canis* *Toxocara cati*	Dogs, cats	Rodents, birds (paratenic hosts)	Cosmopolitan	Visceral *larva migrans*
* Ancylostoma caninum*	Dogs	Rodents	Cosmopolitan	Cutaneous *larva migrans*
* Uncinaria stenocephala*	Dogs (rare in cats)	Rodents	Cosmopolitan	Cutaneous *larva migrans*

The presence of cats and dogs is also associated with gastrointestinal parasites that can be of medical and veterinary importance. Furthermore, these animals can have an important role as spreaders of these parasites through fecal contamination of soil, vegetables and water (Fig. 2). Studies assessing the prevalence of zoonotic gastrointestinal parasites in the feces of stray dogs and cats have constantly pointed out the high number of infected animals with a myriad of parasites pathogenic to humans [46, 48, 93]. This high prevalence is mainly related to the absence of preventative or deworming protocols in feral populations of dogs and cats as well as the availability of infected prey. In addition, most owners are not aware of the possibility of zoonotic parasites from their dogs and cats [40]. Moreover, free-roaming, feral, colonies or house cats with outdoor access, as well as dogs with the same outdoor lifestyle, may defecate in public spaces, increasing the likeability of human exposure [94]. Companion dogs and cats that are allowed to defecate in public places, where feces are not collected or disposed of properly, also represent an important source of environmental contamination [95]. To highlight the impact of dog and cat populations with outdoor lifestyle on the trophic transmission of zoonotic parasitic diseases, examples of the main groups of parasites are given below.

## Not only *Toxoplasma*: zoonotic protozoa that are transmitted by predation

For its pathogenicity during gestation in many animal species, including humans, toxoplasmosis by *T. gondii* [96] is probably one of the most important and better known zoonotic protozoan infections. A wide range of warm-blooded animals are intermediate hosts of this protozoan, while felids are definitive hosts. The biological life cycle in cats is typically perpetuated by their predation of rodents, such as mice (Fig. 3a). For example, in a cat population in southern Poland 68.8% of animals were serologically positive for *T. gondii* with a significantly greater prevalence in older (> 1 year) (83.5%) than in younger cats (48.3%) and in cats kept outdoors than indoors (69.7% vs. 16.7%) [97]. The occurrence of *T. gondii* infection in marine mammals has raised concerns about the role reptiles (e.g. turtles, crocodiles, snakes), amphibians (e.g. frogs, toads) and fish may play as a source of infection [98]. Of the 2988 samples of cold-blooded animals examined in 26 studies reviewed in the literature [98], the number of positive cases of *T. gondii* (*n* = 129) was not sufficient to assess the real involvement of these animal species in the biological life cycle of this protozoan.

Other less studied protozoan diseases are represented by other coccidia (Table 1), for example acute muscular sarcocystosis caused by *Sarcocystis nesbitti* (Eucoccidiorida: Sarcocystidae) [99]. This species of *Sarcocystis*, initially detected in southeast Asia (Malaysia), produces a muscular presentation after the ingestion of sporocysts in food (e.g. uncooked snake meat) or water contaminated with feces from infected definitive hosts (i.e. cats, snakes, humans). This disease has produced a number of recent outbreaks, with > 100 human patients suffering from acute muscular illness on Tioman Island, Malasyia [100], and 89 human patients with molecularly confirmed symptomatic muscular sarcocystosis on Pangkor Island [99]. Most of the cases are associated with the consumption of untreated water [101].

Moreover, the presence of feral cats greatly impacts human and livestock health costs associated with protozoan zoonotic diseases (i.e. toxoplasmosis and human and livestock sarcocystosis) [102]. In Australia, the human costs of these diseases are estimated to be > $6.06 billion Australian dollars per year, and the costs of toxoplasmosis and sarcocystosis affecting sheep and cattle, respectively, are > $11.7 million Australian dollars [102]. Health costs in humans are associated with cats being the definitive hosts of the toxoplasmosis causative agent, thus affecting human health via congenital disease, symptomatic toxoplasmosis and mental health issues.

Dogs and cats may have a role in spreading protozoan species that are associated with environmental contamination, such as *Cryptosporidium* (Eucoccidiorida: Cryptosporidiidae), which causes diarrhea in humans, often leading to outbreaks [103]. *Cryptosporidium parvum* global prevalence in dogs (i.e. 1.28%) [104] was most related to dogs with outdoor lifestyles (i.e. kennel, shelter or stray dogs), which are highly exposed (e.g. 5% in kennel dogs and 1.5% in privately owned dogs from Italy [105]). However, *Cryptosporidium* is globally more prevalent in cats (i.e. 6%) and has a higher prevalence in rural areas and in cats with outdoor lifestyle, associated with livestock and wild animals [106]. Dogs and cats may become infected with zoonotic genotypes of *C. parvum* through fecal-oral transmission or, in some cases, ingesting infected prey [107]. Besides the host-specific *Cryptosporidium felis*, other species have been detected in cats, such as rodent-associated *Cryptosporidium muris* and *Cryptosporidium* rat genotype II, III and IV [108]. However, infection with rodent-associated *Cryptosporidium* spp. in cats may be the result of mechanical transmission due to predation of infected rodents [109]. Hence, future studies to assess the role of predation in the zoonotic transmission and circulation of pathogenic protozoa by dogs and cats are needed.

## Tapeworms in prey: cestode diseases associated to predation

**Fig. 4:**
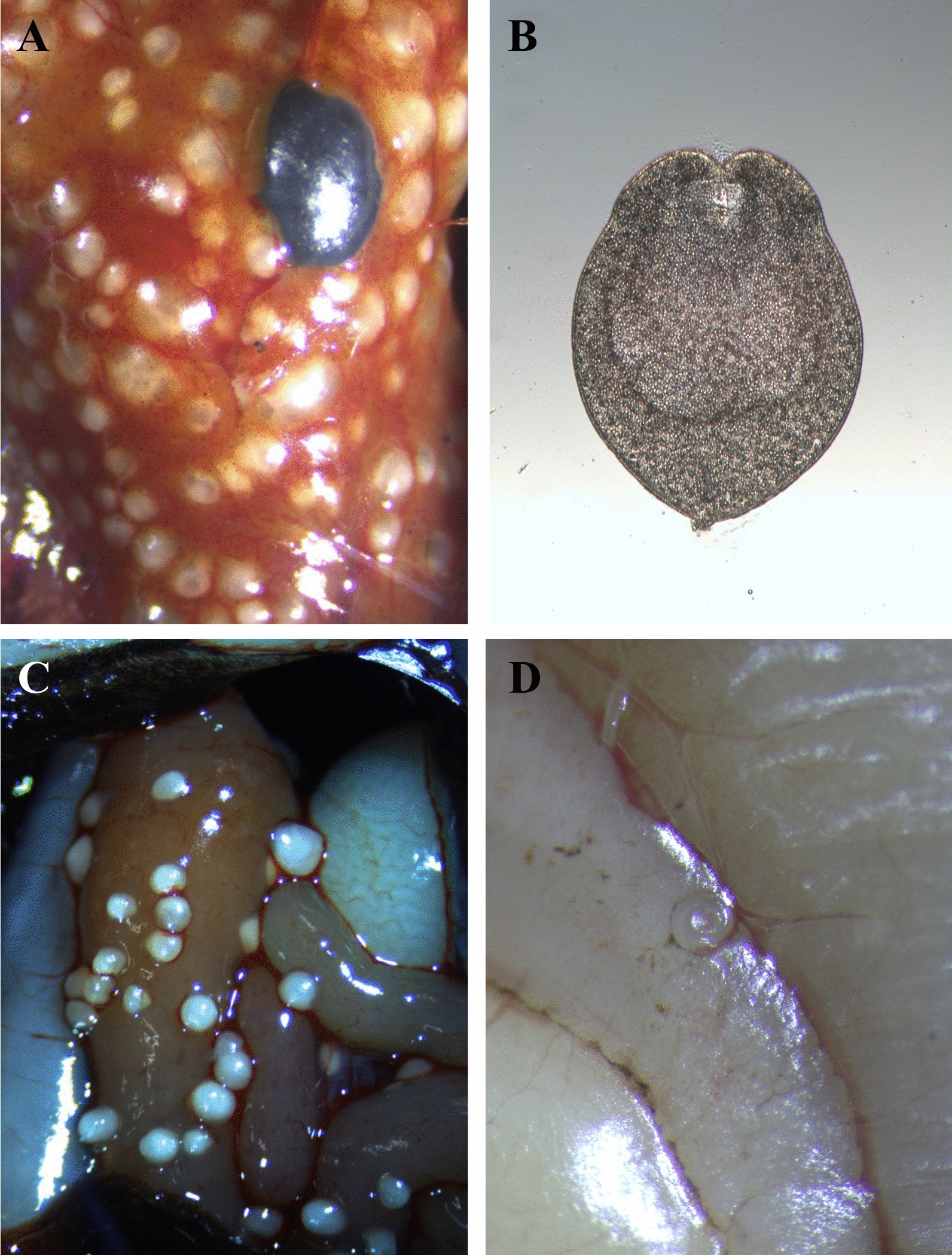
Parasites in intermediate hosts. **A** *Mesocestoides* sp. cysts in liver of lizard; **B** tetrathyridium of *Mesocestoides* sp.; **C** Cyclophyllidea cestode cysts in gecko; **D** nematode larva in the mesentery of a lizard

Four genera of Cyclophyllidea cestods (*Bertiella*, *Dipylidium*, *Raillietina* and *Mesocestoides*) are potentially zoonotic, though less studied and often reported as uncommon findings [110]. In particular, *Dipylidium*, *Mesocestoides* and *Raillietina*, associated with carnivores and rodents, respectively [111], can also have reptiles implicated in their biological life cycles. Indeed, through predation of secondary intermediate hosts (e.g. birds, reptiles and amphibians; Fig. 3b–d), dogs and cats may be infected by *Mesocestoides lineatus* and *Mesocestoides literatus* (Cyclophyllidea: Mesocestoididae), which usually proliferate in their peritoneal cavity as undifferentiated larval stage causing ascites. While adults of these parasites are present in the intestine of carnivores, larval stages perpetuate in two intermediate hosts. The first is probably represented by arthropods (with cysticercoid larvae) and the second by insectivorous vertebrates, harboring elongated larval tetrathyridia (Fig. 4a, b). The identity of the intermediate hosts and biological life cycle remain enigmatic, terrestrial arthropods (e.g. dung beetles, ants, roaches and mites) being considered first intermediate hosts, with the development of metacestode stage following the ingestion of proglottids/oncospheres with the feces of the definitive hosts [112]. Furthermore, the detection of pre-tetrathyridial stages in the body cavity of a ground skink (*Scincella lateralis*) further complicated the understanding of the biology of *Mesocestoides* spp., suggesting that tetrathyridia could develop from hexacanth embryo within a single vertebrate host [113]. Mesocestoidosis is sporadically found in dogs and cats that hunt, typically in rural environments. Human cases of infections have been attributed to *M. lineatus* in Asia and *Mesocestoides variabilis* in North America, though the species identification mainly relies on the geographic origin of these cestodes rather than a clear morphological and/or molecular delineation [110, 114]. In addition, the plasticity of the morphology of larval forms and proglottids and the poor conditions of samples referred by patients hamper a clear understanding of the zoonotic infection routes. Of the 27 human cases of intestinal infections reported by Sapp and Bradbury [110], half are from East Asia (Japan, Korea and China) and the rest from the USA, with one from Rwanda; all were classified as foodborne infections due to the consumption of tetrathyridia in undercooked meat and organs of snakes [110]. Cases from North America, mainly in young children, suggest that these may occur through contact with a variety of exotic pets and geophagy, though these are unlikely a source of tetrathyridia ingestion. In addition, the ingestion of an arthropod first intermediate host (yet unknown) should imply the development of tetrathyridia but not an adult intestinal infection. However, considering that tetrathyridial infections have been documented in non-human primates [115–117], and that in carnivores it is mainly diagnosed in animals during abdominal surgery or necropsy, the absence of cases in humans could be due to the low number of diagnostic opportunities compared to finding scolexes in the patients’ feces. All the above render knowledge about the routes of zoonotic infections and their diagnosis quite complex and enigmatic.

**Fig. 5:**
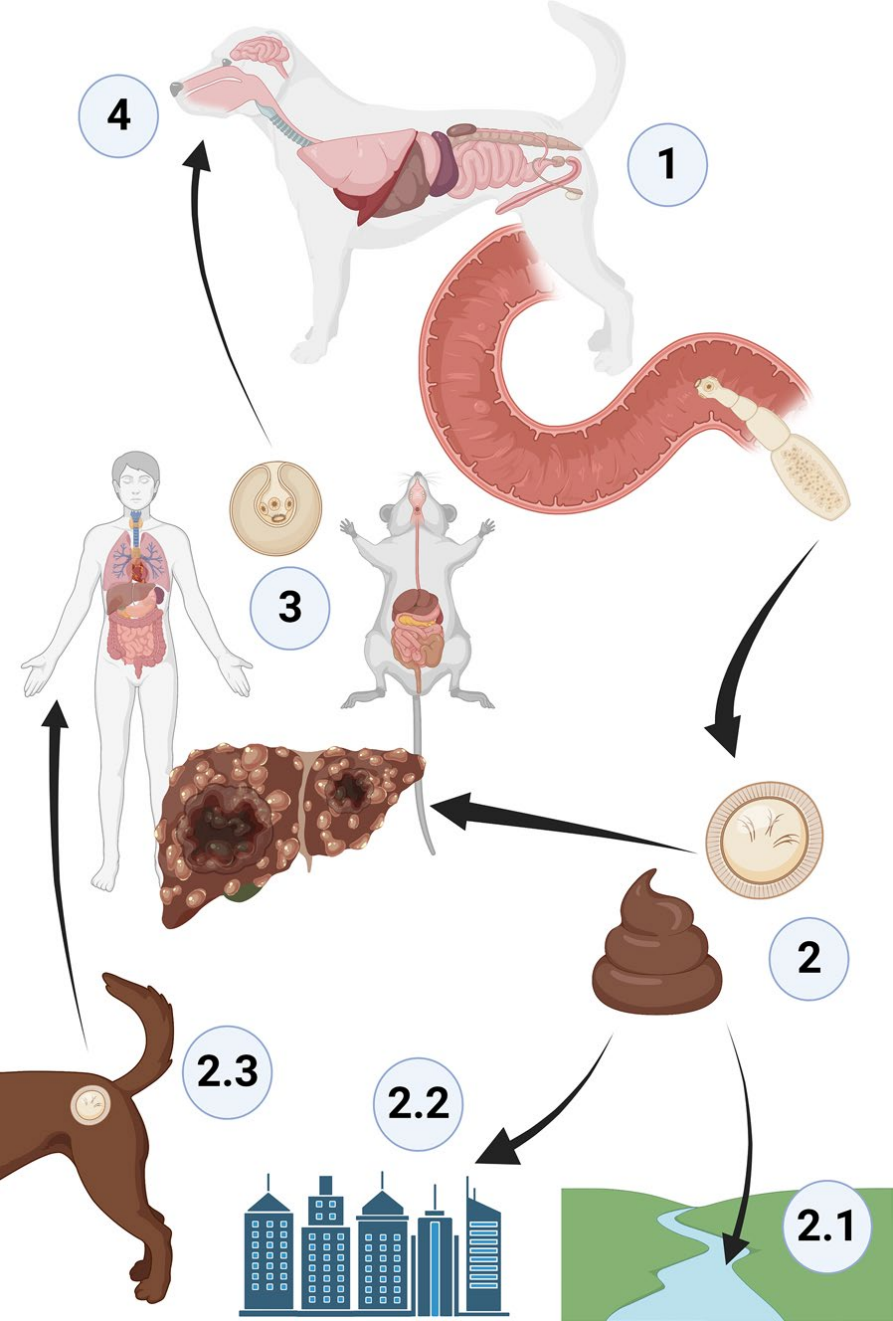
Life cycle of *Echinococcus multilocularis.* (1) Adults develop in intestine of dogs as definitive hosts, (2) eggs are excreted in feces and can contaminate (2.1) water or (2.2) the environment or (2.3) adhere to the fur of definitive hosts. (3) Intermediate hosts (rodents) and humans are infected by ingesting eggs, with larvae developing in cysts in organs (e.g. liver). (4) Dogs ingest intermediate hosts, and larvae develop in adults in the intestine

Furthermore, taeniid cestodes may also be associated with predation as their larval stages depend on an intermediate host (Fig. 4c) [118]. One of the most life-threatening cestode diseases is represented by echinococcosis, with *Echinococcus multilocularis* (Cyclophyllidea: Taeniidae) being strictly associated with predation of small rodents (e.g. voles) by foxes and dogs [119]. Adult cestodes develop on the intestine of canids, which excrete infective eggs in feces that are ingested by intermediate hosts. Humans are infected once in contact with a contaminated environment or eggs adhered to dog fur, causing alveolar echinococcosis (Fig. 5) [120]. This disease is prevalent in the northern hemisphere (e.g. central Europe, the USA, Japan), becoming increasingly present in urban settings [119, 121]. For example, in Switzerland, *E*. *multilocularis* is present in urban areas (i.e. the city of Zurich) because of increasingly synanthropic foxes, serving as definitive hosts, that feed on rodents. In the urban or rural context, free-roaming dogs and cats can become infected by preying on rodents (Fig. 5) [119, 122]. Hence, infected cat and dog populations represent an important zoonotic risk [123]. Indeed, in areas of China where there are large populations of dogs and cats, the risk of infection is higher than that associated with the activity of fox hunting [124].

Other taeniid cestodes associated with lagomorphs and rodents as intermediate hosts, and dogs and cats as definitive hosts, are *Taenia serialis* and *Taenia brauni* (Cyclophyllidea: Taeniidae) [125, 126], which are prevalent in hunting and stray dogs and in cats, in rural areas [126]. Usually, humans become infected after ingesting eggs from the environment, causing subcutaneous or ocular coenurosis, as reported in many cases in Africa [127, 128].

Another group of medical and veterinary important cestodes are the water-associated tapeworms of the genus *Spirometra* (Pseudophyllidea: Diphyllobothriidae), which use dogs, cats and other carnivores as definitive hosts. The most common species found in dogs is *Spirometra mansoni*, and in cats *Spirometra erinaceieuropaei* [129]. Intermediate hosts are represented by aquatic crustaceans (first intermediate hosts, harboring procercoids) and aquatic or semi-aquatic vertebrates, such a fish, amphibians and reptiles (second intermediate hosts, harboring plerocercoids). Both procercoids and plerocercoids (also known as *spargana*) are infective to humans through ingestion of contaminated water, contact or consumption of second intermediate hosts [130, 131]. Sparganosis is frequently reported in Southeast Asia because of common consumption of raw or uncooked snake and frog meat; however, the disease is also present in Africa, the Americas and Australia [128]. A meta-analysis review estimated the global prevalence of *Spirometra* in dogs to be 0.0723%, with *S. mansoni* being the most prevalent species (0.141%) in low-income countries (0.288%) of Africa (0.224%). In the same study, cats presented a higher prevalence (0.1040%), with *S. erinaceieuropaei* as the most prevalent species (0.268%) in lower-middle income countries (0.134%) of Oceania (0.203%) [129, 132].

## Zoonotic nematodes of dogs and cat associated with predation

Many nematodes take advantage of the predator-prey relationship, such as metastrongylids, which use gastropods (i.e. snails and slugs) as intermediate hosts, and a myriad of small animals (i.e. rodents, birds, snakes, lizards) as paratenic hosts (Fig. 4d) [133]. Most of these species (e.g. *Aelurostrongylus abstrusus*, *Troglostrongylus brevior* in cats and *Angiostrongylus vasorum* in dogs) affect only their definitive hosts and have no zoonotic risk [134, 135]. However, zoonotic nematodes of the order Ascaridida (i.e. *Toxocara*) and Strongylida (i.e. *Ancylostoma*, *Uncinaria*) are also associated with the predator-prey relationship, in this case using prey as paratenic hosts [86, 136]. Predation of rodents and birds is important for the completion and maintenance of *Toxocara canis* and *Toxocara cati* (Ascaridida: Toxocaridae), which are worldwide distributed parasites of dogs and foxes, and of cats and other felids, respectively. Toxocariases are regarded as neglected tropical diseases with an overall seroprevalence in the human population of up to 16% [137] and a global population of positive dogs as high as 40% and cats up to 76% [138]. While the infection mainly occurs through the ingestion of larvated eggs from the environment, the predation of paratenic hosts carrying somatic (hypobiotic) L3 represents a major component in the maintenance and perpetuation of the biological life cycles of these zoonotic helminths [139–141]. Though ascarid larvae do not develop in the paratenic hosts, they may survive for up to 10 years, continuing the parasite life cycle for prolonged periods if the paratenic host is consumed by the definitive one [139, 142]. Following the ingestion of larvated eggs by paratenic hosts, larval migration can cause clinical disease (*larva migrans*) depending on the number of larvae and on the organs infected. Paratenic hosts (rodents or birds) harbor larvae in the liver, skeletal muscles or brain tissue, according to host species, with potential consequences on their fitness and behavior. Both *T. canis* and *T. cati* show similar migration patterns toward the central nervous system [143, 144], which may cause disorientation of paratenic hosts, ultimately favoring contact with the definitive hosts. Indeed, *T. canis* cause behavioral alterations and central nervous symptoms (e.g. dullness, somnolence, kyphosis, paresis, incoordination and tremor) in the infected mice, probably because of immune reactions rather than mechanical alterations [141, 145, 146 ]. Overall, these behavioral changes translate into a greater susceptibility to potential predators in the environment. In addition, the infection of the definitive hosts through predation of paratenic hosts has consequences on the time of larval development in adults, which is reduced (about 21 days) since larvae develop directly in the intestine without the liver-trachea-intestine migration route [147]. Also, invertebrates may carry *Toxocara* spp. larvae, including those of *T. cati*, though it is not clear whether these animals act as paratenic hosts or just carriers of hatched larvae in their gut (transport hosts), as already demonstrated for many taeniid eggs [148]. Finally, *T. cati* larvae have been shown to be released from the tissue of *Rumina decollata* snails [149], suggesting a role these mollusks may exert in the transmission of this ascarid through predation. This has been already demonstrated for metastrongylids *A. abstrusus* and *T. brevior* (feline lugworms) with snails, lizards and mice [119], also supported by the coinfections of these two groups of parasites in 18.6% cats from a multicenter European study [150]. In addiotn, Strongylida nematodes (*Ancylostoma* and *Uncinaria*), known as hookworms, cause cutaneous *larva migrans* in humans, dogs and cats, being more prevalent in tropical and subtropical regions [151]. Moreover, *Ancylostoma caninum* (Rhabditida: Ancylostomatidae) can also be associated with eosinophilic enteritis transmitted through the fecal-oral route [152]. Both *A. caninum* and *Uncinaria stenocephala* (Rhabditida: Ancylostomatidae) are usually transmitted orally through the ingestion of third-stage (L3) larvae in the environment or through the skin (percutaneous route) [153] and occasionally through the predation of paratenic hosts represented by rodents [154]. Indeed, it was demonstrated that L3s remain hypobiotic in rodents or other paratenic hosts such as monkeys [151].

## Conclusions

The question of whether cats and dogs should be allowed to wander and have an outdoor lifestyle is an ongoing debate. However, the impact these animals have on the decline and extinction of wildlife populations of small animals (i.e. rodents, birds, reptiles and amphibians) is evident, as is the important role predation has on the transmission of zoonotic parasitic diseases. Three categories of dogs and cats are recognized depending on their contact with humans and healthcare (companion, stray and feral) and, consequently, the risk of preying on potential intermediate hosts of parasites. Although companion animals are more in contact with humans, and less with intermediate hosts, this category represents the major source of zoonotic transmission of parasites by means of fecal-oral infection, environmental contamination and owners’ contact with hunted prey. The main zoonotic parasitic diseases associated with predation are represented by protozoa (toxoplasmosis, acute muscular sarcocystosis, cryptosporidiosis), cestodes (mesocestoidiosis, alveolar echinococcosis, coenusiosis, sparganosis) and nematodes (visceral and cutaneous *larva migrans*). To date, control strategies for dog and cat populations are based on mass sterilization and sheltering of stray animals given that euthanasia and elimination are considered unethical. Thus, there is high permissiveness, mainly in western cultures, toward outdoor lifestyle of dogs and cats, which perpetuates the trophic transmission of zoonotic parasites. Predation, therefore, represents a potential risk for human health that should be addressed by stakeholders and public health officials on different levels (i.e. municipalities, regions, countries). Moreover, conscious and responsible ownership is pivotal for control programs to succeed. This includes education of owners and the community on proper deworming protocols, zoonotic parasites, diminishing outdoor access by offering enriched indoor environments, and collection and hygienic disposal of feces. Thus, it is important that veterinarians advocate for a regular and periodic deworming of all categories of dogs and cats to reduce or clear parasitic burden, eventually reducing environmental contamination. It is important to raise awareness about the zoonotic potential of parasites of dogs and cats associated with predation, not only of owners and veterinarians but also of medical practitioners. As discussed in this review, the trophic transmission of zoonotic parasites has been scarcely studied, given the difficulties in assessing the role of intermediate hosts as well as running field studies to evaluate the risk of transmission. However, future efforts should be performed to address the emergence or re-emergence of cats’ and dogs’ zoonotic parasites that depend on predation to update and improve control strategies. Creating awareness of pet owners, policy makers and scientists regarding the urgent need to reduce predation of intermediate or paratenic hosts by cats and dogs and the risk of zoonotic infection of parasites is ultimately the first step in creating a more conscious society.

## Data Availability

Not applicable.
